# Long-Term Outcome Over 15 Years of Prosthesis for Thumb Carpometacarpal Joint Arthritis: A Case Series

**DOI:** 10.7759/cureus.73480

**Published:** 2024-11-11

**Authors:** Takanori Shintaku, Masanori Nakayama, Hideaki Ishii, Mitsuru Yagi, Hiroyasu Ikegami

**Affiliations:** 1 Orthopaedic Surgery, Toho University Ohashi Medical Center, Tokyo, JPN; 2 Orthopaedic Surgery, International University of Health and Welfare (IUHW), Narita, JPN

**Keywords:** arthroplasty, implant, long-term outcomes, prosthesis, thumb carpometacarpal joint arthritis

## Abstract

This report presents the long-term (over 15 years) results of four Japanese patients who underwent total joint replacement for thumb carpometacarpal (CMC) joint arthritis. Four patients (mean age 60.5 years) underwent prosthesis replacement for thumb CMC joint arthritis with AVANTA(TM) (Small Bone Innovations, Morrisville, PA, USA) implants between 2001 and 2004, with a mean follow-up of 20 years and seven months. One patient was classified as Eaton stage 2 and three patients were classified as Eaton stage 3 prior to surgery. Although radiographs at the last follow-up showed loosening of the trapezium-side implant in all cases, three of the patients were excellent and only one was good according to Eaton's clinical criteria, and none of them reported severe pain or significant problems. Despite radiographic evidence of implant loosening and subsidence, long-term results were positive with minimal clinical symptoms. The results suggest that thumb CMC joint arthroplasty can provide satisfactory long-term results. However, the use of thumb CMC joint prostheses is currently limited in Japan, and alternative surgical methods such as suspension arthroplasty are more common. We believe that the thumb CMC joint prosthesis is as effective as any other surgical method based on the good results we have seen.

## Introduction

Thumb carpometacarpal (CMC) joint arthritis is one of the most frequently encountered hand diseases in outpatient clinics, with a prevalence of 50.2% in Japan [1] because the CMC joint is loaded 12 times more than the phalanges during thumb pinch and is susceptible to osteoarthritis [2]. Thumb CMC joint arthritis is more common in postmenopausal women [3], who present with pain and swelling at the base of the thumb that interferes with activities of daily living, including household tasks. When the joint is refractory to orthotics or other conservative treatments, surgery is performed to relieve pain, restore a stable, mobile, and strong thumb, and achieve long-term stability [4,5]. Numerous surgical procedures are performed, including suspension arthroplasty, arthrodesis, and prosthesis [5]. The choice of surgical procedures varies widely from region to region. Especially in North America, suspension arthroplasty is mainly performed due to its good results. In contrast, the results of prostheses are reported to be poor, and the survey results show that the frequency of suspension arthroplasty is increasing every year [6]. However, prostheses are preferred as an effective treatment in Europe because of their good postoperative results [7]. The thumb CMC joint has the specific shape of a plyometric joint and is classified as a biaxial joint. AVANTA(TM) (Small Bone Innovations, Morrisville, PA, USA) implants are a resurfacing prosthesis that can reproduce a structure very close to the shape of the thumb CMC joint and can preserve the trapezium, which is subject to axial pressure [8] and were the implants used in the surgery we performed.

To the best of our knowledge, no literature on the long-term results of thumb CMC joint prostheses in Japanese patients has been published in English. We report the long-term results of four Japanese patients with thumb CMC joint prostheses over 15 years.

## Case presentation

The details of the four patients who underwent surgery between 2001 and 2004 and were followed up for more than 15 years are shown in Table 1. The mean age at surgery was 60.5 years, and the mean follow-up period was 20 years and seven months. AVANTA(TM) (Small Bone Innovations, Morrisville, PA, USA) implants were used in all cases.

**Table 1:** Patient details. E, Excellent; G, Good.

Case No.	Age at surgery	Preoperative X-ray evaluation based on Eaton's classification	Surgical side	Follow-up term (Y: years, M: months)	X-ray evaluation at the final observation		Final pinch power (kg) (surgical side/contralateral side)	Eaton’s clinical criteria
					Implant loosening	Implant subsidence		
1	59	3	L	19Y2M	+	-	4.0/3.0	E
2	60	3	L	21Y0M	+	-	3.5/2.5	E
3	61	2	R	21Y9M	+	+	4.0/1.0	E
4	62	3	L	20Y3M	+	+	4.5/5.0	G

**Figure 1:**
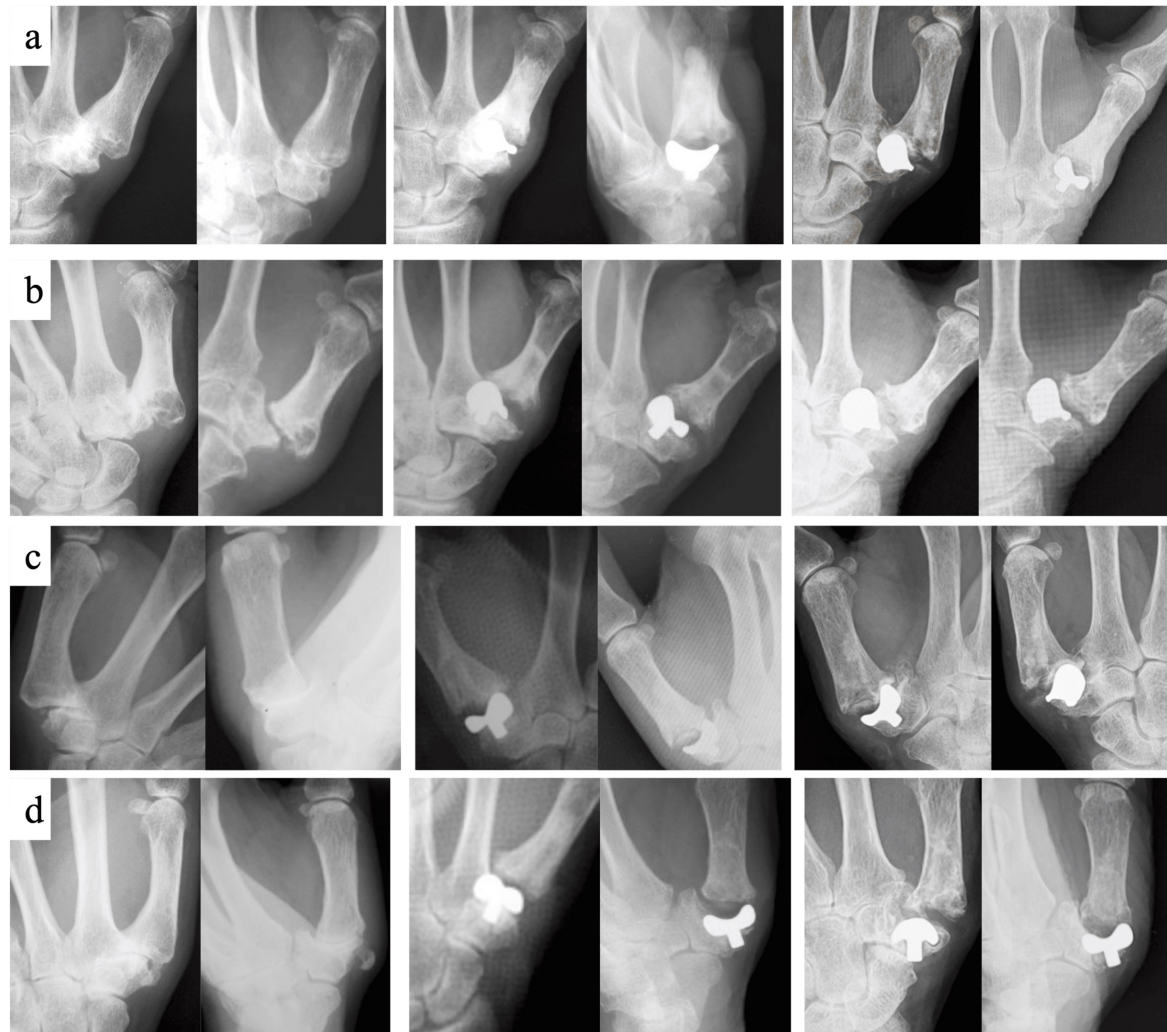
Postoperative radiographs (a: case 1, b: case 2, c: case 3, d: case 4): (left) preoperative, (middle) immediate postoperative, and (right) last observation (frontal and oblique views, respectively).

Two hand surgeons evaluated the patients’ radiographic findings. In disagreements regarding radiographic findings between the two surgeons, the final assessment was determined after discussion. According to Eaton’s classification in 1984 [4], preoperative radiographic evaluation revealed one case with stage 2 and three cases with stage 3. Radiographs taken immediately postoperatively and at the last follow-up are shown in Figure 1.

Although two cases showed implant subsidence on the trapezium side and all cases showed implant loosening on the final radiograph, none of the patients complained of pain or other complicated symptoms. The clinical evaluation at the final observation was based on Eaton’s clinical criteria (Table 2) [4], which was excellent in three cases and good in one case.

**Table 2:** Eaton’s clinical criteria [4]. Failures were not improved from preoperative status.

Eaton's clinical criteria in 1984; results were classified as excellent, good, fair, or failure	
Excellent	No pain, pinch strength more than 90% of the contralateral thumb, and no instability
Good	Occasional pain after prolonged activity, pinch strength greater than 70% of the contralateral thumb, and minimum laxity
Fair	Frequent pain with average use, strength less than 70% of the contralateral thumb, or mild joint laxity but were better than preoperatively

Our four cases are presented here. Case 1 was a 59-year-old woman who underwent surgery. Based on preoperative radiographs, the patient was diagnosed with stage 3 Eaton’s classification. Radiographs of the surgical site at 19 years and two months postoperatively showed some subsidence of the implant on the trapezium side and mild dislocation or subluxation of the joint. The tip pinch force was comparable to the contralateral side, and the outcome based on Eaton’s clinical criteria was excellent. Case 2 was a 59-year-old woman. Based on preoperative radiographs, the patient was diagnosed with stage 3 Eaton’s classification. Radiographs of the surgical site at 21 years postoperatively showed some subsidence of the implant on the trapezium side. The tip pinch force was comparable to the contralateral side, and the outcome based on Eaton’s clinical criteria was excellent. Case 3 was a 61-year-old woman. Based on preoperative radiographs, the patient was diagnosed with stage 2 Eaton’s classification. Radiographs of the surgical site at 21 years and nine months postoperatively showed some loosening and subsidence of the implant on the trapezium side. The tip pinch force of the operative side was much higher than the contralateral side because the nonoperative side of the CMC joint of the thumb was painful due to osteoarthritis and the pinch force of the nonoperative side was low. Case 4 was a 62-year-old woman. The patient complained of CMC joint pain and was diagnosed with stage 3 Eaton’s classification based on preoperative radiographs. Radiographs of the surgical site 20 years and three months postoperatively showed significant subsidence and loosening of the implant on the trapezium side and mild dislocation or subluxation of the joint. The tip pinch force was comparable to the contralateral side, and the outcome according to Eaton’s clinical criteria was good.

## Discussion

The thumb CMC joint is potentially unstable, which is one of the reasons for its susceptibility to osteoarthritis [9]. The current mainstream prostheses for thumb CMC joint arthritis in Europe are “double-mobile” types with mobility on both the metacarpal bone and trapezium sides [10]. Although one of the main complications of thumb CMC joint prostheses is loosening of the trapezium implant and not the metacarpal implant, “double-mobile” implants have the advantage of improving joint laxity and placing less mechanical stress on the trapezium compared to simple prostheses (arthroplasty of the trapezium side only, or surface replacement of both the metacarpal bone and the trapezium), resulting in less loosening and better long-term results. In our study, there were no revision cases, even though our implants were only surface replacements for both metacarpal and trapezium sides, and not double-mobile types. This may be due to the ingenuity of the surgical procedure, particularly the improvement in joint stability with soft tissue reconstruction in addition to the prosthesis. Instability of the thumb CMC joint is mainly due to traction from the abductor pollicis longus (APL) [11]. Therefore, we used the dorsal approach in all cases, and after replacing the articular surfaces with prostheses, to achieve joint stability, the APL tendons are detached and reattached to the metacarpal base in an advanced position after implant placement in our surgical procedure.

According to a worldwide survey of 1138 hand surgeons, there were significant differences in the number of implant prostheses between the United States and Europe, with European hand surgeons reporting far higher numbers [12]. The long-term outcome of a thumb CMC joint prosthesis is generally considered to be poor [13,14]; therefore, the number of prostheses for thumb CMC joint arthritis has decreased, especially in North America, and non-implant arthroplasty, especially suspension arthroplasty, is now a major surgical procedure [15]. In contrast, several reports have compared prostheses with suspension arthroplasty for thumb CMC joint arthritis and found no difference in clinical outcomes and complications between the two [16,17]. A Norwegian registry study reported good results with thumb CMC joint prosthesis, with an overall 10-year survival rate of 90% [18]. Furthermore, according to a systematic review [17,19], the poor long-term outcome of thumb CMC joint prostheses was due to technical errors, such as implant malpositioning or poor indication with respect to the shape or bone quality of the trapezium. 

In our four cases, the radiographic change at the last observation showed that the implants on the trapezium side had loosened in all patients, and two of them had sunk; however, only one patient complained of symptoms at the last observation, and it was mild. In addition, the final pinch force of the surgical side was more than 70% of that of the contralateral side in all cases. Since the previous reports of implant arthroplasty for thumb CMC joint arthritis showed a low correlation between radiographic and clinical symptoms [20], which is similar to our results, implant loosening or subsidence may not contribute to the long-term outcome. Unfortunately, at the time of writing this manuscript, implants for the thumb CMC joint have been commercially discontinued in Japan, only a few custom-made joint prostheses have been invented and used by some surgeons, and performing implant arthroplasty for the thumb CMC joint is now unrealistic for Japanese hand surgeons. Although osteotomy, arthrodesis, and non-implant arthroplasty are performed depending on the condition of each case, the optimal technique has not yet been determined. We believe that the results of this study may help to provide evidence that implant arthroplasty for thumb CMC joint arthritis is as good as other surgical methods.

This study has several limitations. First, this study is retrospective and lacks a control group. Second, although we were able to summarize the cases that could be followed up long-term, we did not include cases that dropped out during the study (it is possible that the cases with poor outcomes were mixed in the dropped-out cases). Third, patients with good clinical outcomes in the early postoperative period were more likely to be followed for a long period, i.e., there may be a selection bias. Finally, since this was only a single-center study and only a limited number of surgeons were involved in all the cases, our results may not be generalizable to all cases of thumb CMC joint arthritis.

## Conclusions

Our cases with implant arthroplasty for the thumb CMC joint arthritis have showen that loosening or subsidence on radiographic findings after surgery are not always symptomatic and do not require revision, and they may not have a significant impact on clinical outcomes. Therefore, based on our cases with good long-term results, we believe that implant arthroplasty for thumb CMC joint arthritis with appropriate procedures of surrounding soft tissues may be a good surgical option similar to other methods.
